# A Rare Presentation of Undifferentiated Pleomorphic Sarcoma in the Subpectoral Space

**DOI:** 10.7759/cureus.44482

**Published:** 2023-08-31

**Authors:** Kylee Arthurs, Barbara S Suening, Elisabeth Barrar, Husain Abbas, Steve Webb

**Affiliations:** 1 Medicine, Orange Park Medical Center, Jacksonville, USA; 2 Medicine, Edward Via College of Osteopathic Medicine, Spartanburg, USA; 3 General Surgery, HCA Florida Orange Park Medical Center, Orange Park, USA; 4 Advanced and Bariatric Surgery, Jacksonville Memorial Hospital, Jacksonville, USA; 5 General Surgery, HCA Florida Memorial Hospital, Jacksonville, USA

**Keywords:** neoplasm, engorged veins, tumor, sarcoma, undifferentiated pleomorphic sarcoma

## Abstract

Soft tissue sarcomas (STS) are often described as asymptomatic, rapidly expanding masses, particularly in the extremities or trunk. Undifferentiated pleomorphic sarcoma (UPS), a high-grade variant of STS, ranks as the second most prevalent subtype in the United States. It predominantly affects males between their fifth and seventh decades. Its often benign symptomatology, however, can lead to initial misdiagnosis and subsequent mismanagement. We present the case of a 57-year-old Caucasian male, previously in good health, who experienced a recurring subpectoral lesion causing discomfort and mass-related effects. Initial management included incision and drainage, which provided temporary relief. The biopsy revealed a diagnosis of grade 3 UPS. The lesion's recurrence two months later was accompanied by local invasion into adjacent skin and musculature as well as metastasis to the right hemiliver. A comprehensive understanding of UPS among medical professionals is vital for accurate diagnosis and facilitating prompt intervention to prevent avoidable complications and optimize patient outcomes.

## Introduction

With age, cancer diagnoses become more prevalent. Sarcoma, a less common cancer type, constitutes roughly 1% of adult malignancies [1]. Among the various subtypes, undifferentiated pleomorphic sarcoma (UPS) stands as a prominent diagnosis, accounting for 17.1% of cases. Its incidence predominantly occurs in Caucasian men between their fifth and seventh decades of life, frequently originating in the extremities and occasionally in the chest wall or trunk [2]. Surgical excision with wide margins, along with adjuvant chemotherapy and radiation, are common treatment options. In the absence of adequate surgical margins, this type of malignancy is prone to local recurrence and can metastasize with larger, deeper primary tumor dimensions [2]. Metastasis typically occurs in the lungs (90%) and less frequently in the bones (8%) [3]. Risk factors for UPS are typically linked to sociodemographic characteristics, such as age and race, along with a history of prior radiation to the affected area [2,4].

The etiology of UPS remains a topic of ongoing discussion. Recent investigations have revealed that the cells originate from a mesenchymal lineage, bearing a close resemblance to mesenchymal stem cells [5,6]. Genetic factors contributing to this malignancy have also been explored, with several gene mutations proposed as potential triggers for primary tumor development. Clinical trials involving gene-specific and tumor marker-targeted chemotherapeutic agents for UPS management have also been conducted. However, due to the early stage of these studies, further research is warranted for the optimization of patient outcomes [3,5].

## Case presentation

A previously healthy 57-year-old Caucasian male presented to the emergency department due to a painful, right-sided chest wall mass that progressively enlarged over one year. He worked in lawn care and attributed the lesion's growth to the trauma of his backpack rubbing against his chest. The lump was drained a few months earlier while he was overseas and was noted to contain red-tinged fluid. Since his return, the mass continued to grow and has caused significant pain and numbness over his right chest and right shoulder. He denied having fever, chills, night sweats, drainage, and unintentional weight loss. Family history was contributory for non-Hodgkin's lymphoma in his mother, which was diagnosed in her late 70s.

Physical examination revealed a large, firm, immobile mass in the anterior right chest wall associated with engorged vessels. There were no signs of infection, overlying skin changes, or palpable lymphadenopathy. The range of motion in his right upper extremity was within normal limits.

**Figure 1:**
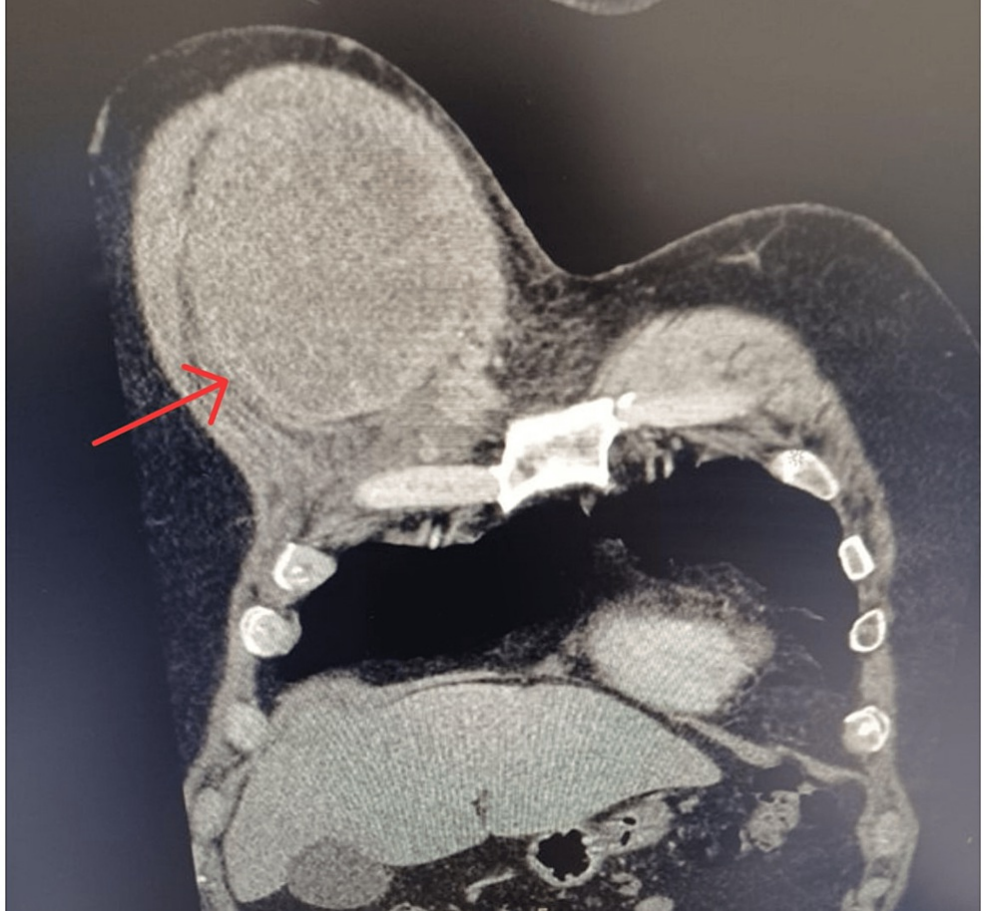
Coronal view (deep) of heterogenous mass measuring 13.7 cm x 8.6 cm x 11.5 cm in the right subpectoral space without signs of lymphadenopathy

**Figure 2:**
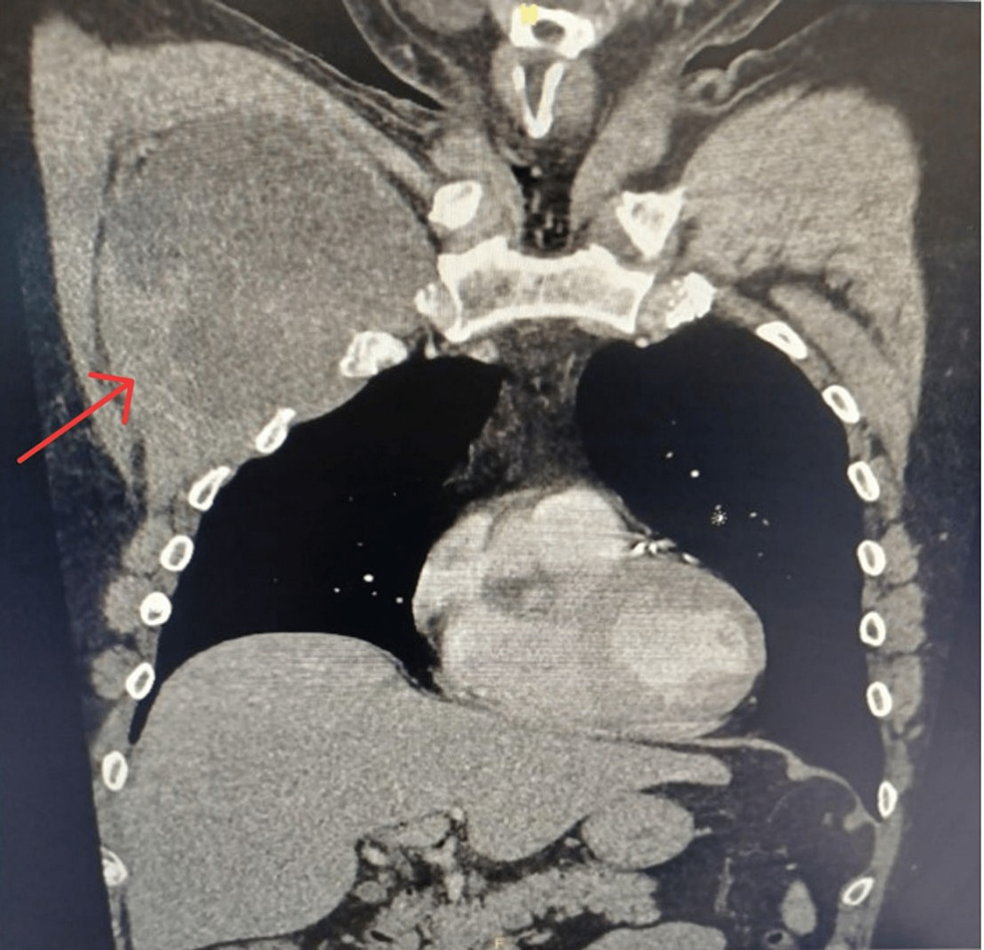
Coronal view (superficial) of heterogenous mass measuring 13.7 cm x 8.6 cm x 11.5 cm in the right subpectoral space without signs of lymphadenopathy

**Figure 3:**
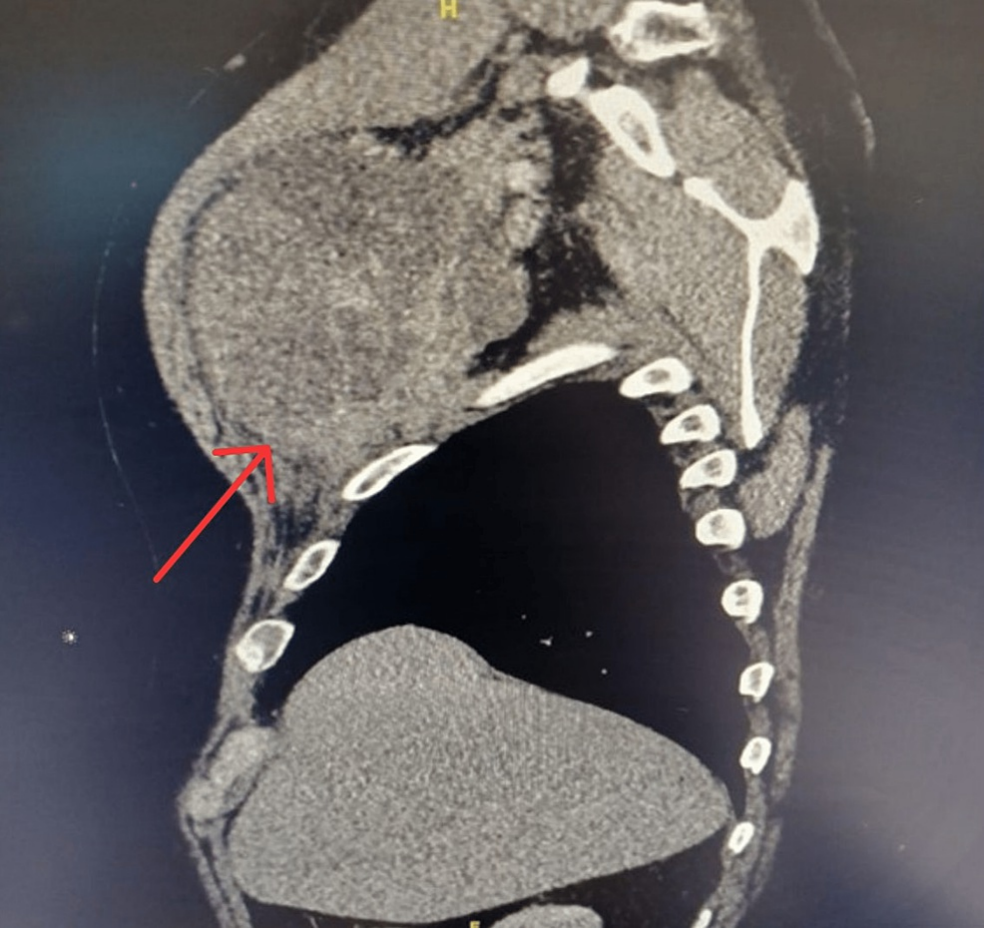
Sagittal view of heterogenous mass measuring 13.7 cm x 8.6 cm x 11.5 cm in the right subpectoral space without signs of lymphadenopathy, inflammatory processes, or fat stranding

**Figure 4:**
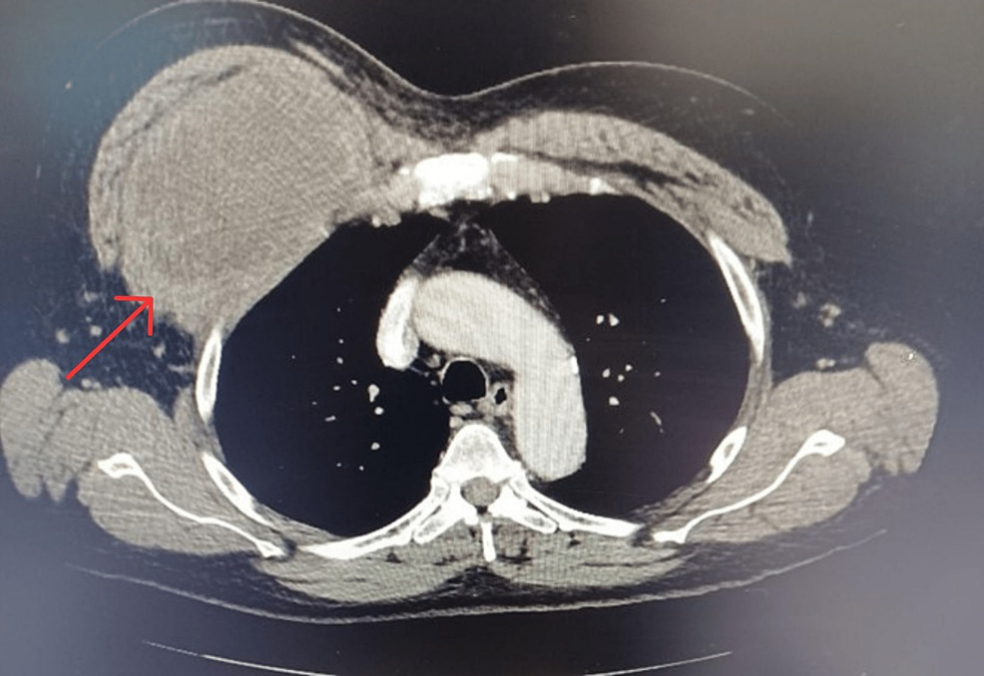
Axial view of heterogenous mass measuring 13.7 cm x 8.6 cm x 11.5 cm in the right subpectoral space without signs of lymphadenopathy, inflammatory processes, or fat stranding

A computerized tomography (CT) scan of the chest demonstrated a heterogeneous mass measuring 13.7 cm x 8.6 cm x 11.5 cm under the right pectoral muscle, with no associated adenopathy or signs of inflammation (Figures 1-4). There was no evidence of invasion into surrounding tissues. Of note, a 13 mm area of low density was appreciated in his right hepatic lobe.

After discussing treatment options with a multidisciplinary team and the patient, surgical intervention was chosen over preoperative biopsy due to the patient's discomfort with the tumor. The procedure involved draining the right subpectoral chest wall mass, which revealed sanguineous fluid, blood clots, and necrotic tissue. The capsule was removed with 1 cm margins, and results from pathology indicated a grade 3 undifferentiated pleomorphic sarcoma with a positive vimentin stain.

Following surgery, the patient began treatment with doxorubicin and cisplatin. He returned to the emergency department two months later due to a syncopal episode at the infusion center. Assessment at that time revealed recurrence of the right retropectoral mass, measuring 9.3 cm x 14.1 cm x 12.8 cm, with invasion into surrounding musculature and skin. Additionally, a hypodense mass, measuring 2.1 cm x 2.5 cm, was detected in the posterior aspect of the right hemiliver. The patient was referred to oncology for further management. He completed six cycles of chemotherapy before he was lost to follow-up.

## Discussion

UPS often masquerades benign conditions, leading to potential misdiagnoses and mismanagement. Recognizing this rare condition is crucial for timely and effective treatment. As its exact pathogenesis remains unclear, tailored therapeutic options are limited [7]. Recent research has indicated that tumorigenesis in UPS may originate from side population (SP) cells, which are capable of self-renewal, growth, and proliferation. SP cells derive their name from their tendency to fall to the "side" during flow cytometry, distinguishing them from the main cell population. These SP cells exhibit elevated Hedgehog/Notch pathway activity, which is suggestive of higher-grade, dedifferentiated sarcomas [8]. Another theory on UPS pathogenesis pertains to ABC transporter proteins, which have the capacity to expel drugs from cells and contribute to chemotherapy resistance [8]. Given the incomplete understanding of UPS's underlying causes, treatment strategies rely on current approaches to other sarcomas.

The staging of UPS utilizes the tumor, node, and metastasis (TNM) system [9]. Grading considers differentiation, mitotic count, and tumor necrosis, following French Federation of Cancer System (FNCLCC) criteria [9]. The FNCLCC scale is thought to be more effective in predicting distant metastasis and morbidity [10,11]. The approach to staging and treatment are outlined in Table 1. Current systemic therapies involve anthracycline-based regimens, specifically doxorubicin, ifosfamide, and mesna [9]. Recurrence was associated with advanced age and inadequate surgical margins. The five- and 10-year overall survival rates were 60% and 48%, respectively [12]. Larger tumor sizes (>10 cm) were associated with up to a seven-fold increase in mortality rate [12].

**Table 1:** FNCLCC staging and treatment FNCLCC: French Federation of Cancer System.

FNCLCC scale	Description	Treatment
Stage 1	Tumor ≤ 5 cm, ≤ 15 cm without lymph node involvement or metastasis, or grade 1	Wide local excision with ≥2 cm margins
Stage 2	Tumor ≤ 5 cm without lymph node involvement or metastasis, or grade 2/3	Neoadjuvant/adjuvant radiation and surgical resection
Stage 3	Tumor > 5 cm without lymph node involvement or metastasis, or grade 2/3	Neoadjuvant/adjuvant chemoradiation and surgical resection
Stage 4	Any size cancer with lymph node spread, with/without metastasis, of any grade	Select stage 4: Neoadjuvant/adjuvant chemoradiation and surgical resection

The current chemotherapeutic regimen for UPS involves doxorubicin, ifosfamide, and mesna, which focuses on inducing cellular damage through DNA alkylation and free radical formation [13,14]. However, the clinical use of these agents is marred by their adverse effects, such as cardiotoxicity and hemorrhagic cystitis [13,14]. Present investigations into gamma-secretase inhibitors (GSIs), a distinct class of drugs, have revealed their potential to inhibit Notch pathways, a likely contributor to UPS tumorigenesis, although their efficacy in reducing tumor size has exhibited variable outcomes. However, GSIs exhibit decreased toxicity, demonstrating a more favorable safety profile [15,16]. Further exploration of therapeutic modalities for UPS is imperative as it holds the potential to augment overall survival rates and mitigate metastatic disease or local recurrence, which are critical challenges in the management of this complex disease.

## Conclusions

UPS is an uncommon, high-grade STS associated with a poor prognosis. The tendency to omit UPS from the differential diagnosis, often due to STS being of low order in the differential diagnosis tree, commonly results in diagnostic oversights. Educating healthcare providers about this aggressive and challenging-to-identify cancer is crucial to ensure timely and appropriate patient care, leading to improved treatment outcomes. Furthermore, additional research into UPS genetics and molecular pathways is necessary for the advancement of tailored chemotherapeutic options.
